# AVS-referenced diagnostic performance of ^68^Ga-Pentixafor PET/CT for detecting unilateral disease in primary aldosteronism: a systematic review and meta-analysis

**DOI:** 10.3389/fendo.2026.1777128

**Published:** 2026-08-19

**Authors:** Junjie Su, Changliang Chi, Shuaikang Wang, Ge Bian, Dong Xing, Ming Zhang, Mingyang Chang, Jiayi Yin, Xiaoqing Wang

**Affiliations:** Department of Urology, First Hospital of Jilin University, Chaoyang District, Changchun, Jilin, China

**Keywords:** 68Ga-Pentixafor PET/CT, Adrenal vein sampling, Diagnostic performance, primary aldosteronism, Sensitivity, specificity, Subtype classification, unilateral lesions

## Abstract

**​Background:**

Differentiation of unilateral from bilateral primary aldosteronism (PA) is crucial for identifying patients who are likely to be cured by adrenalectomy. This review evaluated the adrenal vein sampling (AVS)-referenced performance of ^68^Ga-Pentixafor positron emission tomography/computed tomography (PET/CT) for detecting unilateral aldosterone-producing lesions.

**Methods:**

We searched PubMed, Web of Science, and Embase for relevant articles published up to May 2025. This review followed a prespecified population, index test, comparator, outcome, and study design (PICOS) framework. Using Meta-DiSc v.1.4 and Stata software, sensitivity, specificity, likelihood ratios, and diagnostic odds ratios were pooled.

**Results:**

Twelve studies met the inclusion criteria. Hu et al., 2023 was retained only for qualitative description and excluded from the primary quantitative synthesis because of its clear outlying influence; thus, 11 studies were included in the main pooled synthesis. The pooled sensitivity and specificity were 0.75 (95% CI: 0.67–0.82) and 0.81 (95% CI: 0.74–0.87), respectively. The positive and negative likelihood ratios were 4.05 and 0.31, respectively. The diagnostic odds ratio (DOR) was 12.59 (95% CI: 7.21–22.01).

**Conclusion:**

^68^Ga-Pentixafor PET/CT showed fair concordance with AVS-based lateralization and may have a role as an adjunctive test in selected clinical scenarios, especially when AVS is unavailable, non-diagnostic, or technically demanding. Further prospective trials with validation against postoperative biochemical and clinical outcomes are warranted.

**Systematic Review Registration:**

https://www.crd.york.ac.uk/PROSPERO/view/CRD420251067457, identifier CRD420251067457.

## Highlights

- Primary aldosteronism (PA) requires accurate identification of unilateral disease to select patients who may benefit from curative adrenalectomy.
- This systematic review and meta-analysis shows that ^68^Ga-Pentixafor PET/CT has good diagnostic performance for detecting unilateral PA compared with AVS-based classification.
- ^68^Ga-Pentixafor PET/CT may serve as a non-invasive adjunct to AVS and improve diagnostic workflows when AVS is unavailable or technically challenging.

## Introduction

Primary aldosteronism (PA) is the predominant etiology of secondary endocrine hypertension, and a precise differentiation between unilateral and bilateral forms is essential for effective treatment direction (1–4). Patients with unilateral adrenal adenoma are best treated with curative surgery, whereas treatment of patients with idiopathic hyperaldosteronism (IHA) is often medical. Bilateral PA is usually treated medically, whereas unilateral adrenalectomy for patients with unilateral PA has consistently achieved better clinical remission rates compared with medical treatment (5–9). Adrenal vein sampling (AVS) is currently considered the reference standard for subtype differentiation of patients with PA (10). Despite its efficacy, AVS is invasive, costly, technically demanding, and mainly available in experienced centers (5, 11–13). These limitations stress the immediate need for an accurate, safe, reproducible, and cost-effective non-invasive method for differentiating PA subtypes that separate unilateral from bilateral disease.

Targeting the chemokine receptor CXCR4 with the radiolabeled ligand ^68^Ga-Pentixafor is a promising molecular imaging approach, because CXCR4 is upregulated in aldosterone-producing tissues and is linked to CYP11B2 expression (14). This association was reported by Heinze et al. (15). Recently, the ligand Pentixafor labeled with the positron-emitting isotope ^68^Ga has been developed as a PET tracer for subtype differentiation in patients with PA. ^68^Ga-Pentixafor PET/CT in PA subtyping might have diagnostic value, as suggested by recently published studies (16–18). The ability of this imaging method to meaningfully distinguish unilateral aldosterone excess is uncertain. This study evaluated the AVS-referenced diagnostic performance of ^68^Ga-Pentixafor PET/CT in detecting unilateral primary aldosteronism using AVS as the reference standard. By reviewing and analyzing the existing literature, we sought to elucidate the possible therapeutic implications and clinical utility of the new imaging method as an adjunct to AVS in the diagnostic workup of PA.

## Methods

### Search strategy

We conducted this systematic review and meta-analysis according to the Preferred Reporting Items for Systematic Reviews and Meta-Analyses (PRISMA) 2020 statement and a predefined PICOS framework. By conducting extensive searches in three major biomedical databases, namely PubMed, Embase and Web of Science, relevant papers were systematically collected. In order to improve the sensitivity and guarantee a wide range of covered literature, the search strategy combined both the controlled vocabulary terms (Medical Subject Headings, MeSH) and free-text keywords. The key search terms used were “primary aldosteronism”, “^68^Ga-Pentixafor PET/CT”, “adrenal vein sampling” and “lateralization”. No limitations were set on the language of publication, the country of publication or the status of the publication. The literature search included articles published up to May 2025.

### Eligibility criteria

The eligible studies for the analysis were those which met the following conditions: (1) the studies involved patients of any sex or age with a definite clinical diagnosis of primary aldosteronism; (2) the sample size should be at least 5 patients; and (3) the full-text articles should provide sufficient raw diagnostic data. To ensure the uniformity of the methods and avoid possible selection bias, the studies were excluded if they satisfied one of the following points: (a) less than five participants were enrolled in either the case or control group; (b) there was insufficient information to construct diagnostic contingency tables; or (c) the articles were not available in full-text form or did not contain essential diagnostic data. Furthermore, articles categorized as non-original clinical research, such as reviews, meta-analyses, conference abstracts, case reports, animal experiments, and *in vitro* or mechanistic investigations, were excluded. Duplicate publications from the same research cohort or authors were detected and eliminated. Furthermore, studies that only assessed AVS or ^68^Ga-Pentixafor PET/CT, without direct comparison or validation of lateralization accuracy, were omitted from further analysis.

### Reference standard and index test definitions

Data from included studies were used to construct 2 × 2 contingency tables comprising true positives (TP), false positives (FP), true negatives (TN), and false negatives (FN), with AVS-confirmed lateralization serving as the reference standard. Diagnostic 2 × 2 tables were constructed using study-specific PET/CT criteria rather than a unified maximum standardized uptake value (SUVmax) threshold. AVS protocols, including the use of adrenocorticotropic hormone (ACTH) or cosyntropin stimulation, sampling method, selectivity index, and lateralization index cut-off values, were extracted when reported and summarized in **Table 1**. PET/CT positivity was extracted according to the prespecified diagnostic criteria reported in each original study, including visual lateralization, SUVmax-based criteria, SUVmax lateralization index, lesion-to-liver ratio, or lesion-to-normal adrenal ratio when available. Study-level AVS-positive and PET/CT-positive lateralization counts were calculated from the extracted 

**Table 1:** Study characteristics, adrenal imaging findings, lateralization counts, and diagnostic criteria used for AVS and ^68^Ga-Pentixafor PET/CT.

Study	Year/country	Design/population	N	CT-detected adrenal findings/population	AVS-positive unilateral disease, n	PET/CT-positive unilateral disease, n	Reference standard	AVS protocol/cut-off	PET/CT positive criterion	SUVmax/ratio criterion	2×2 data (TP/FP/FN/TN)	Analysis note
Jinbo Hu et al.	2023/China	Diagnostic study; patients with PA undergoing PET/CT and AVS; 43 UPA and 57 BPA reported.	100	NR	43	33	AVS	AVS-based lateralization; detailed SI/LI cut-offs NR.	PET/CT subtyping based on adrenal uptake lateralization.	SUVmax-LI at 10 min; cut-off 1.65.	33/0/10/57	Qualitative synthesis only; excluded from primary pooled analysis as an outlier.
Yanqing Zheng et al.	2023/China	Prospective single-center study; patients with PA or nonfunctioning adenoma.	5	PA or nonfunctioning adrenal adenoma; exact CT nodule count NR.	2	3	AVS-based lateralization used for this meta-analysis; surgery/pathology/follow-up reported in the original study.	SI ≥2 without ACTH; SI ≥3 after ACTH; LI used for dominant-side assessment.	Visual assessment: adrenal nodule uptake higher than contralateral and adjacent adrenal tissue.	SUVmax, LLR and LAR measured; thresholds reported according to lesion size.	2/1/0/2	Included in primary pooled analysis.
Jie Ding et al.	2024/China	Prospective enrollment; PA patients with adrenal micronodules <1 cm on CT.	34	Adrenal micronodules <1 cm on CT.	15	15	AVS-based lateralization used for this meta-analysis; surgery/pathology/follow-up reported in the original study.	AVS performed without ACTH stimulation; detailed calculation methods reported in the original study/supplement.	Visual assessment followed by semiquantitative analysis.	SUVmax, LLR, LAR and SUVmax-LI calculated; study-specific thresholds used.	10/5/5/14	Included in primary pooled analysis.
Rui Zuo et al.	2024/China	Retrospective single-center study; PA patients with bilateral adrenal lesions on CT.	32	Bilateral adrenal lesions on CT.	13	15	AVS	AVS performed; corticotropin stimulation reported in some AVS procedures; detailed SI/LI cut-offs NR.	PET lateralization based on 10-min and 40-min imaging results and bilateral adrenal uptake.	Dominant-side SUVmax and bilateral SUVmax ratio assessed; no universal SUVmax cut-off applied.	10/5/3/14	Included in primary pooled analysis.
Xiangshuang Zhang et al.	2024/China	Retrospective study; PA patients with PET/CT and classification diagnosis of UPA/BPA.	208	NR	128	94	AVS-based lateralization used for this meta-analysis; surgery/pathology reported in the original study.	Sequential AVS; saline or corticotropin infusion; SI ≥2 without corticotropin and SI ≥5 with corticotropin; LI used for subtype classification.	Visual analysis: uptake higher than adjacent and contralateral adrenal tissue.	SUVmax-LI and dominant-side adrenal-to-liver ratio assessed; study-specific criteria used.	87/7/41/73	Included in primary pooled analysis.
Xuan Yin et al.	2024/China	Prospective cohort; PA patients undergoing PET/CT and AVS; histology/follow-up also reported.	26	NR	15	17	AVS-based lateralization used for this meta-analysis; histology/follow-up reported in the original study.	AVS performed after medication adjustment; detailed AVS criteria reported in the original study.	Visual analysis: uptake higher than ipsilateral or contralateral normal adrenal tissue.	SUVmax, LCR and LLR assessed; reported cut-offs included SUVmax 5.71, LCR 1.39 and LLR 3.05.	13/4/2/7	Included in primary pooled analysis.
Guoyang Zheng et al.	2025/China	Prospective surgical management cohort; PA patients receiving PET/CT-guided surgical treatment.	14	NR	9	8	AVS-based lateralization used for this meta-analysis; postoperative outcomes reported in the original study.	AVS used to identify endocrine dominance; detailed SI/LI cut-offs NR.	Visual and semiquantitative analysis; positive/dominant side used for surgical guidance.	SUVmax reported; median SUVmax 10.2; no universal cut-off for this meta-analysis.	6/2/3/3	Included in primary pooled analysis.
Nan Lu et al.	2025/China	Clinical cohort comparing PET/CT with AVS; pathology/follow-up assessed after adrenalectomy.	45	NR	38	28	AVS-based lateralization used for this meta-analysis; pathology/follow-up reported in the original study.	All patients underwent AVS; AVS success rate 98.0%; detailed SI/LI cut-offs NR.	Visual analysis of PET/CT lateralization; SUVmax also evaluated.	SUVmax cut-off 11.95 for APA lateralization; SUVmax cut-off 5.85 in all PA patients.	28/0/10/7	Included in primary pooled analysis.
Rui Zuo et al. (dual-time)	2025/China	Retrospective dual-time PET/CT study; PA patients with lesions ≥1 cm and <1 cm analyzed.	136	PA patients with adrenal lesions ≥1 cm and <1 cm analyzed.	87	62	AVS-based lateralization used for this meta-analysis; postoperative follow-up reported in the original study.	AVS completed with PET/CT in 136 patients; detailed SI/LI cut-offs NR.	Dual-time 10-min and 40-min visual/semiquantitative PET/CT interpretation.	APA cut-off: 10-min LAR 1.95; AVS-based subtyping: 10-min SUVmax-LI 1.46.	52/10/35/39	Included in primary pooled analysis.
Rui Zuo et al. (unilateral nodules)	2025/China	Retrospective study; PA patients with unilateral adrenal nodular lesions on CT.	52	Unilateral adrenal nodular lesions on CT.	42	28	AVS	AVS used for lateralization; detailed SI/LI cut-offs NR.	Visual and semiquantitative analysis.	10-min SUVmax >8.17 for UPA; additional lesion-size-specific criteria reported.	27/1/15/9	Included in primary pooled analysis.
Tieci Yi et al.	2025/China	Prospective cohort; PA patients with bilateral or unilateral adrenal masses.	37	Bilateral or unilateral adrenal masses.	28	27	AVS	Non-ACTH-stimulated sequential AVS; SI >2 for successful catheterization; LI used for lateralization.	Comprehensive analysis including visual assessment and bilateral adrenal SUVmax.	SUVmax cut-off 6.86; SUV ratio cut-off 2.40; visual analysis showed best performance.	25/2/3/7	Included in primary pooled analysis.
Wen-Cai Zheng et al.	2025/China	Prospective cohort with randomized surgical allocation among eligible patients.	90	Adrenal lesion-positive surgical allocation cohort; exact CT nodule count NR.	49	50	AVS-based lateralization used for this meta-analysis; postoperative PASO outcomes reported in the original study.	AVS performed by experienced interventional radiologists; detailed criteria reported in the original study.	Adrenal lesion positive if uptake was higher than corresponding normal adrenal tissue.	SUVmax used to predict clinical complete success; optimal outcome cut-off 9.8.	43/7/6/34	Included in primary pooled analysis.

ACTH, adrenocorticotropic hormone; APA, aldosterone-producing adenoma; AVS, adrenal vein sampling; BPA, bilateral primary aldosteronism; CT, computed tomography; FN, false negative; FP, false positive; LAR, lesion-to-normal adrenal ratio; LCR, lesion-to-contralateral adrenal ratio; LI, lateralization index; LLR, lesion-to-liver ratio; NR, not reported; PASO, Primary Aldosteronism Surgical Outcome; PA, primary aldosteronism; PET/CT, positron emission tomography/computed tomography; SI, selectivity index; SUVmax, maximum standardized uptake value; SUVmax-LI, SUVmax lateralization index; TN, true negative; TP, true positive; UPA, unilateral primary aldosteronism. AVS-positive unilateral disease was calculated as TP + FN, and PET/CT-positive unilateral disease was calculated as TP + FP. CT-detected adrenal findings are reported as described in each original study; NR indicates that the exact CT-detected adrenal nodule count was not consistently reported in the available extracted data.

### Study selection

Literature screening and reference management were done by using EndNote to arrange the obtained data systematically. A three-stage standard process was carried out separately to enhance the methodological quality. In the first stage, duplicate records were deleted. The titles and abstracts were chosen by the research team according to the given inclusion and exclusion criteria to select the potentially appropriate studies. In the second stage, the full texts of the remaining studies were collected and checked carefully to decide their eligibility and to gather the diagnostic performance data. If there was any disagreement between the two evaluators, it was settled by discussion and, if required, the opinion of a third evaluator was taken to prevent bias and ensure the consistency in selecting the studies.

### Data extraction

Baseline characteristics, AVS protocols, PET/CT positivity criteria, and diagnostic 2 × 2 data, including true positives, false positives, false negatives, and true negatives, were extracted independently by two reviewers. Discrepancies were resolved by discussion or, when necessary, consultation with a third reviewer.

### Risk of bias assessment

The methodological quality of the included studies was evaluated using the Quality Assessment of Diagnostic Accuracy Studies-2 (QUADAS-2) tool, which examines four domains: patient selection, index test, reference standard, and flow and timing (19). Each domain was assessed individually and was classified as exhibiting low, high, or unclear risk of bias (19).

### Statistical analysis

Diagnostic performance evaluations were performed using Stata software (StataCorp, College Station, TX, USA) and Meta-DiSc v.1.4 (20, 21). Threshold effects were assessed to determine whether discrepancies in diagnostic performance were due to varying cut-off values across trials. Spearman’s rank correlation coefficient was used to evaluate the relationship between sensitivity and specificity. A p-value <0.05, together with a shoulder-like or asymmetrical configuration on the summary receiver operating characteristic (SROC) curve, was suggestive of a threshold effect (20).

Pooled estimates of sensitivity, specificity, positive likelihood ratio (PLR), negative likelihood ratio (NLR), and diagnostic odds ratio (DOR) were computed in the absence of substantial threshold effects. The area under the summary receiver operating characteristic curve (AUC) was employed to evaluate the overall diagnostic performance. A high AUC value suggests better discriminative ability. The heterogeneity among the studies was examined using Cochran’s Q test and quantified using the I² statistic (22, 23). Considering the expected clinical and methodological heterogeneity across studies, including differences in AVS protocols and PET/CT positivity criteria, random-effects models were used for the main pooled diagnostic performance estimates. The clinical relevance of ⁶⁸Ga-Pentixafor PET/CT was also investigated using Fagan's nomogram, which estimates post-test probabilities to assist clinical interpretation and decision-making. A sensitivity analysis was performed by excluding the study with an obviously different diagnostic odds ratio to assess the robustness of the pooled results.

## Results

**Figure 1:**
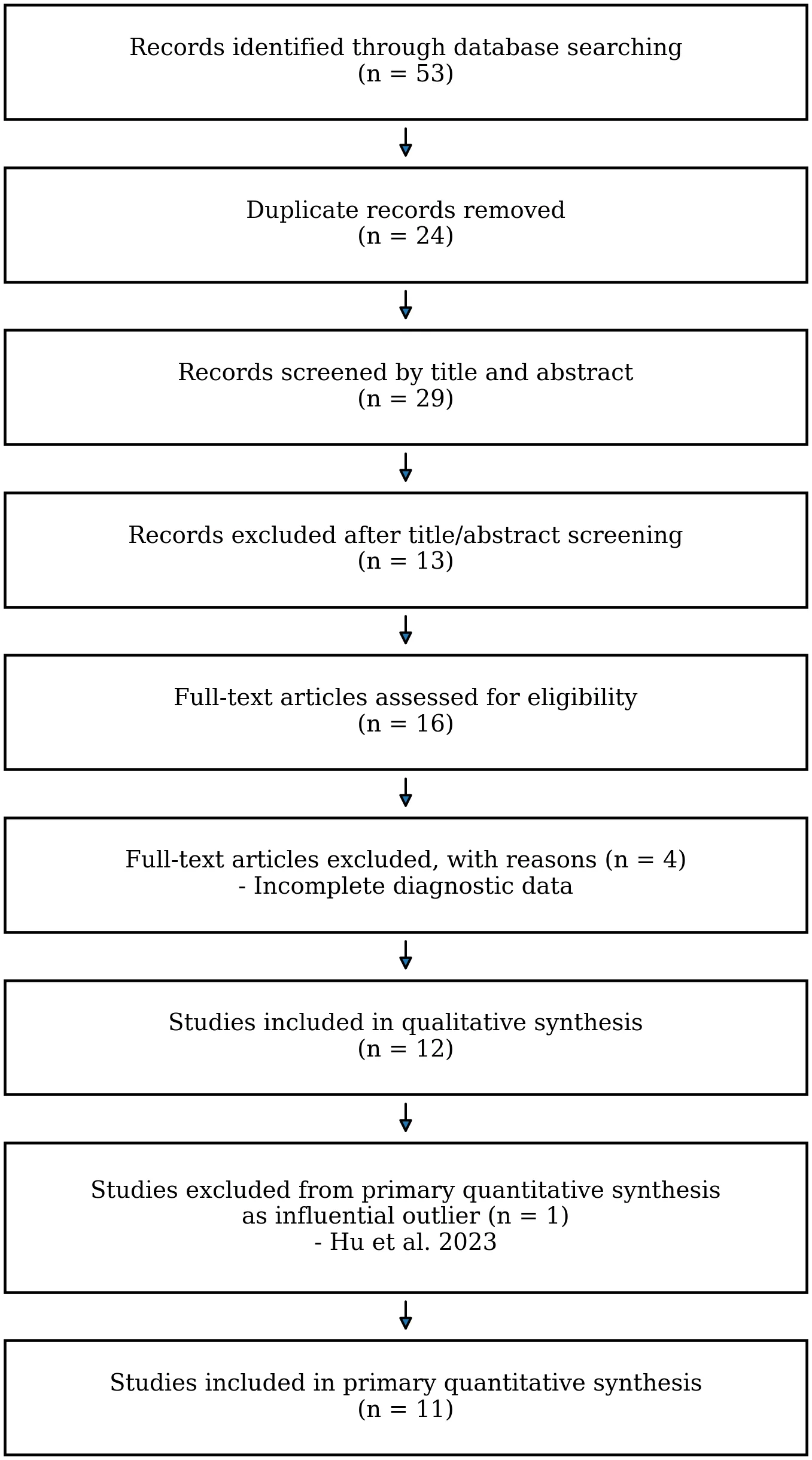
PRISMA flow diagram.

A total of 24 duplicate studies were excluded, followed by the exclusion of an additional 13 studies that did not meet the inclusion criteria, resulting in 16 studies remaining for the final analysis. At this stage, four studies were excluded due to the absence of the complete diagnostic data. As a result, 12 studies met all inclusion criteria. After exclusion of Hu et al. in 2023 (17) as an outlier, 11 studies were included in the primary pooled analysis (**Figure 1**) (17, 18, 24–33).

All included studies were original research articles of moderate-to-high methodological quality, each providing sufficient data for quantitative synthesis. Risk-of-bias assessment using the QUADAS-2 tool indicated a generally low risk of bias across domains. Specifically, the “Patient Selection” domain demonstrated the highest methodological rigor, since most studies clearly defined their inclusion and exclusion criteria and enrolled participants consecutively or prospectively, thus reducing the likelihood of selection bias.

### Heterogeneity and threshold effect

Analysis of threshold effects using Meta-DiSc v.1.4 revealed no significant threshold phenomenon (20). The Spearman’s correlation coefficient between logit-transformed sensitivity and 1–specificity was 0.132 (p = 0.684), and the SROC curve exhibited a symmetric shape without a shoulder-arm pattern, both indicating the absence of threshold effects. Overall heterogeneity was assessed using Cochran’s Q and *I²* statistics. The initial Q statistic was 17.88 with 11 degrees of freedom (P= 0.084), and the *I*² was 38.5%, suggesting moderate heterogeneity. Tau² was estimated at 0.3792.

Following its exclusion, the Spearman’s correlation coefficient remained statistically non-significant (r = 0.139, P = 0.683). In the Stata restricted maximum likelihood (REML) model for the DOR analysis, heterogeneity was acceptable (Q = 12.21, df = 10, P = 0.271; I² = 29.59%; T² = 0.24). These findings suggested that exclusion of this study reduced between-study heterogeneity.

### Publication bias

**Figure 2:**
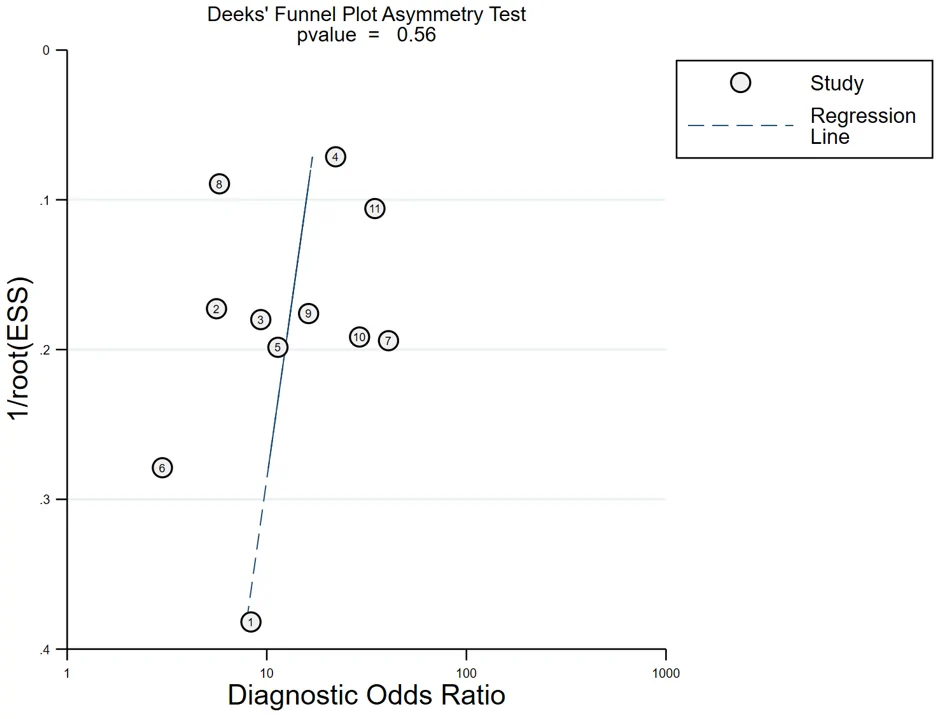
Deeks' funnel plot for the assessment of potential publication bias.

Deeks’ funnel plot was constructed using Stata software to evaluate possible publication bias. The resulting p-value was 0.56, exceeding the 0.10 threshold for statistical significance, suggesting no apparent risk of publication bias among the included studies (**Figure 2**).

### Pooled diagnostic performance

**Figure 3:**
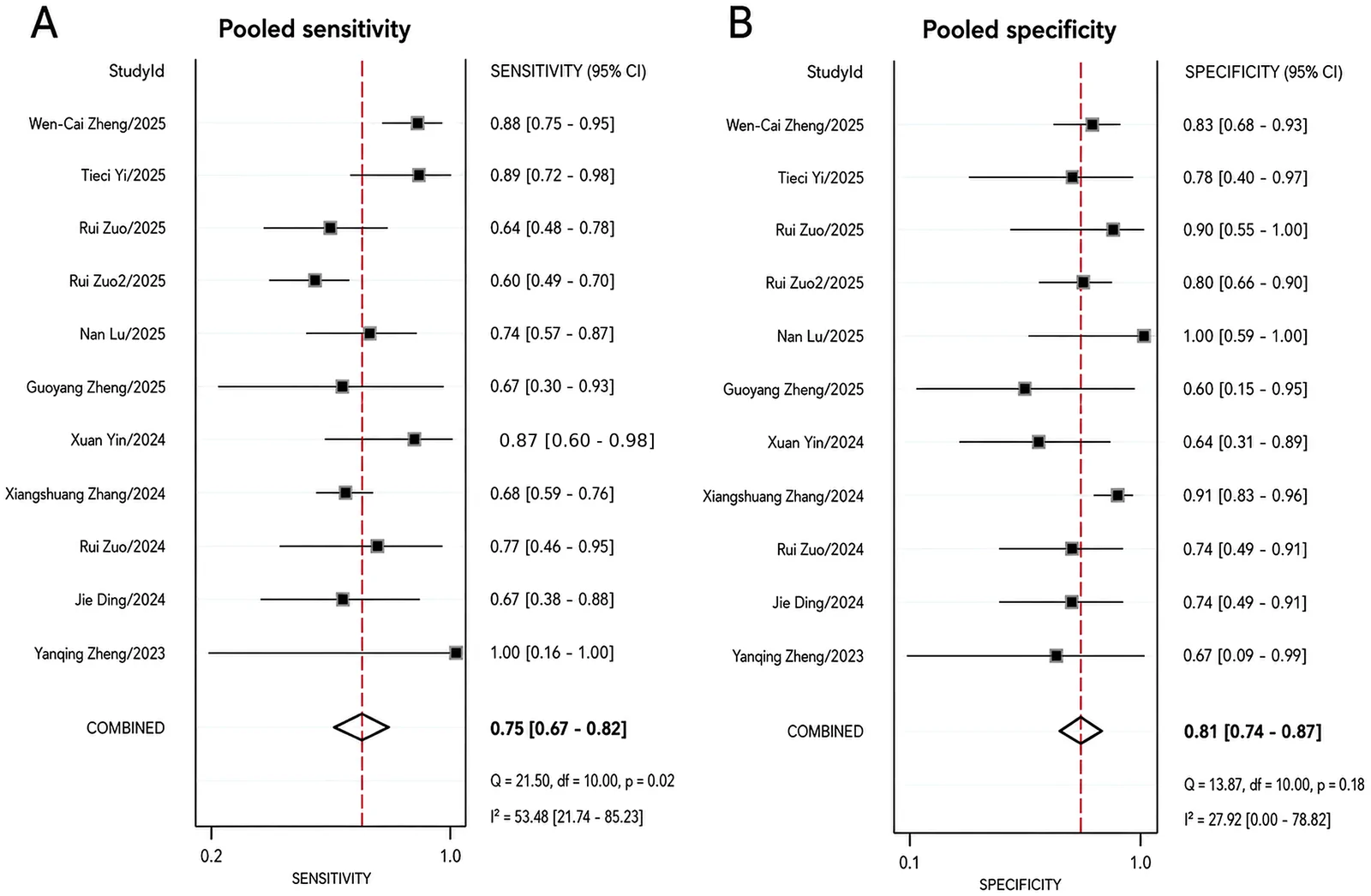
Pooled sensitivity and specificity of ^68^Ga-Pentixafor PET/CT for detecting unilateral disease in patients with primary aldosteronism. **(A)** pooled sensitivity. **(B)** pooled specificity.

**Figure 4:**
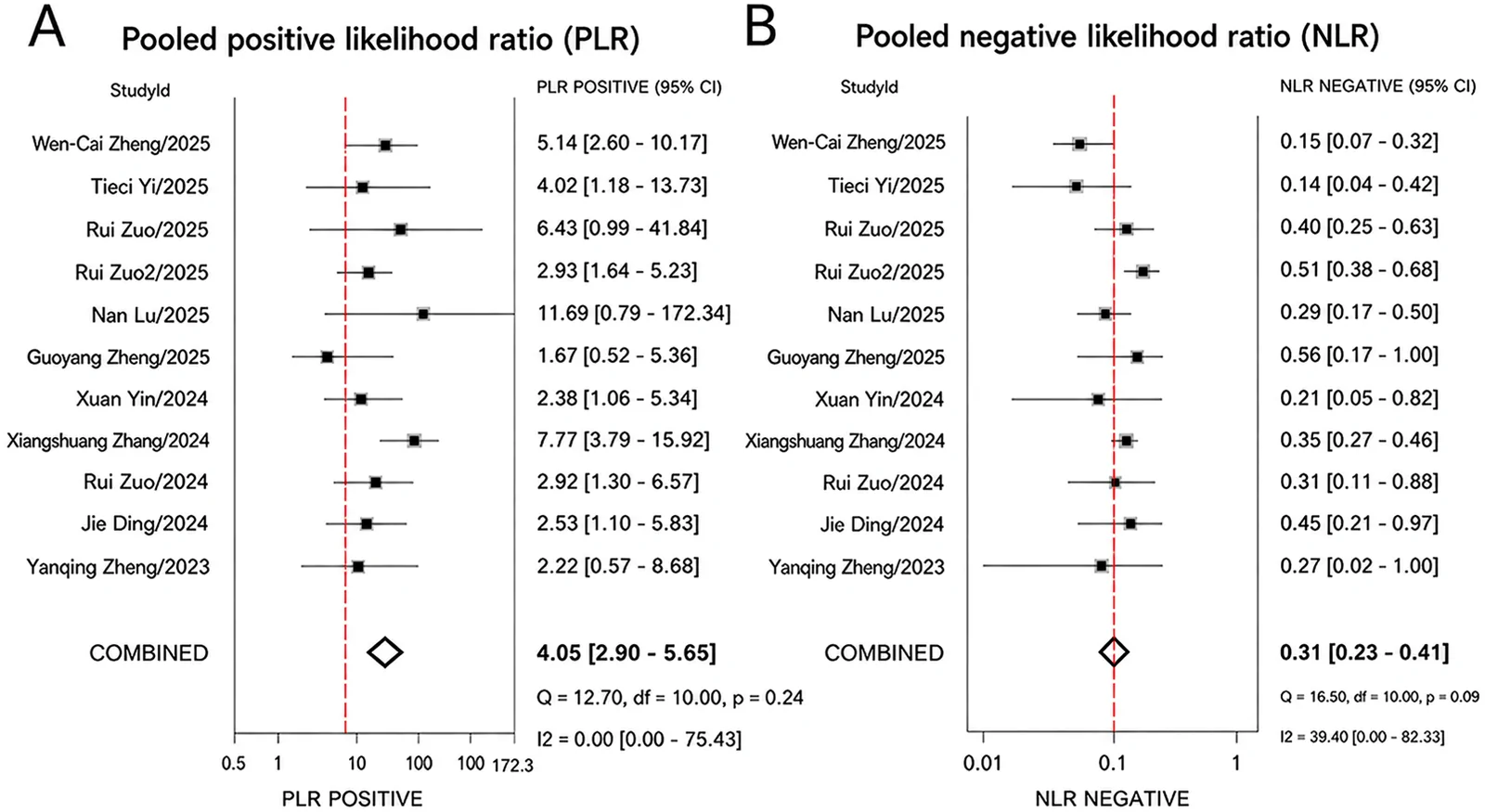
Pooled positive likelihood ratio (PLR) and negative likelihood ratio (NLR) of ^68^Ga-Pentixafor PET/CT. **(A)** pooled PLR. **(B)** pooled NLR.

**Figure 5:**
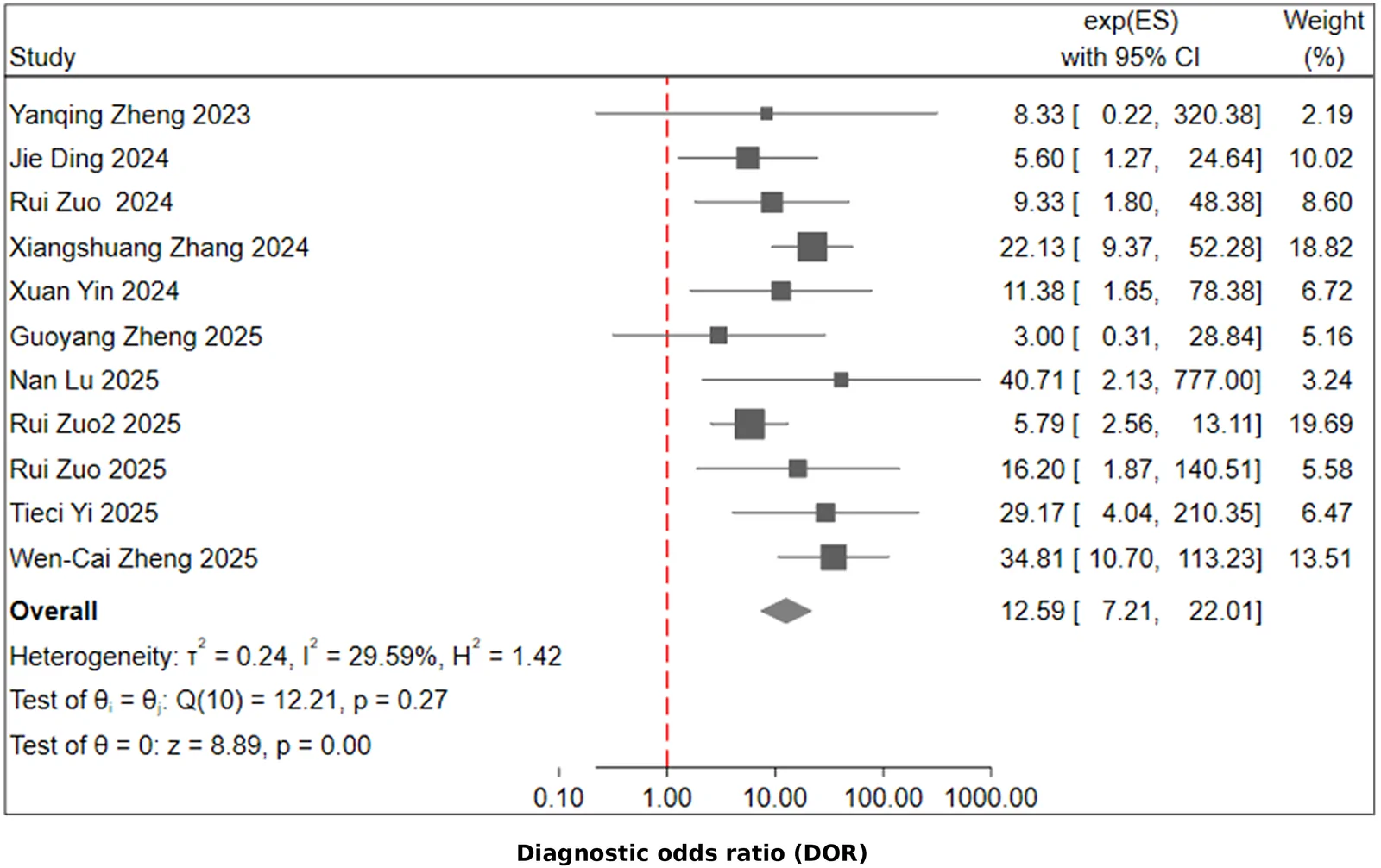
Forest plot of the pooled diagnostic odds ratio (DOR) of ^68^Ga-Pentixafor PET/CT for identifying unilateral disease.

**Figure 6:**
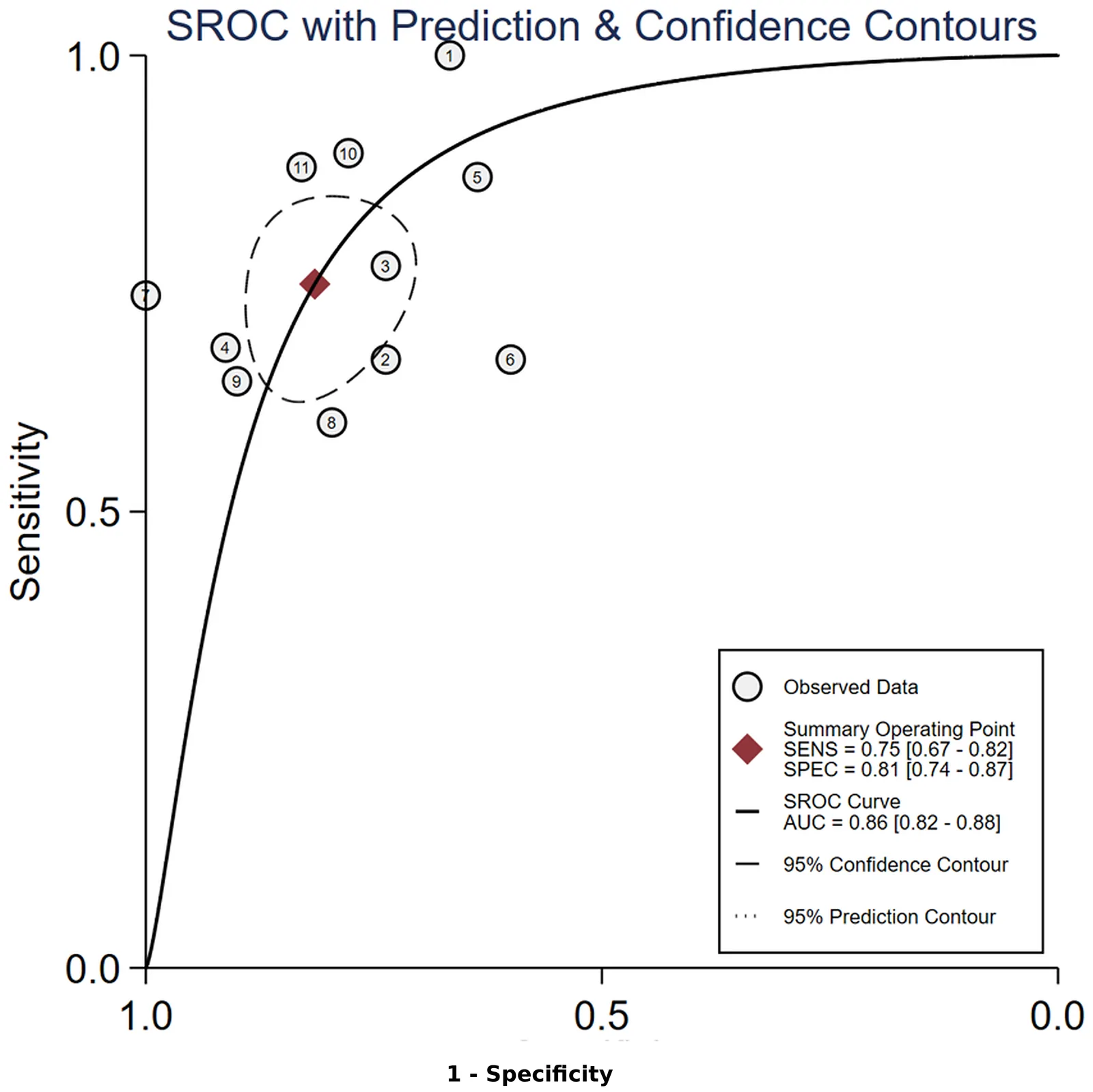
Summary receiver operating characteristic (SROC) curve of ^68^Ga-Pentixafor PET/CT for identifying unilateral primary aldosteronism.

**Figure 7:**
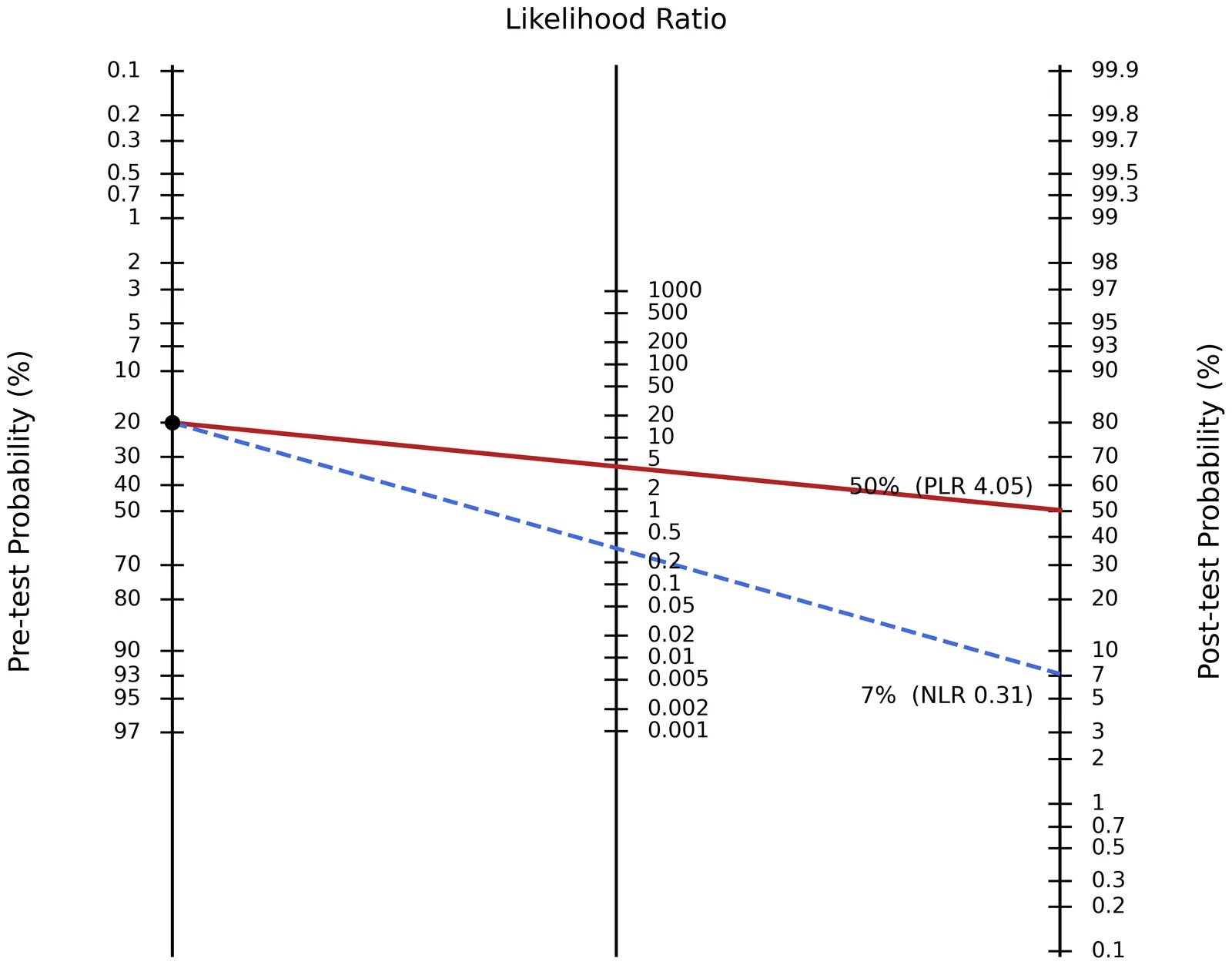
Fagan’s nomogram illustrating the post-test diagnostic probability based on the diagnostic performance of ^68^Ga-Pentixafor PET/CT in identifying unilateral lesions in primary aldosteronism.

The diagnostic performance of ^68^Ga-Pentixafor PET/CT to detect unilateral aldosterone-producing lesions in individuals with PA was good compared with AVS-based lateralization. The diagnostic tool had a sensitivity of 0.75 (95% CI: 0.67–0.82) and a specificity of 0.81 (95% CI: 0.74–0.87), indicating good ability to detect unilateral disease and reduce false-positive classification of bilateral disease as unilateral disease (**Figure 3**). The PLR was 4.05 (95% CI: 2.90–5.65), indicating an elevation in post-test probability after a positive result. The NLR was 0.31 (95% CI: 0.23–0.41), indicating an ability to reduce the probability of unilateral PA when the test result was negative (**Figure 4**). The DOR was 12.59 (95% CI: 7.21–22.01), indicating good discriminative capability (**Figure 5**). The area under the SROC curve (AUC) was 0.86, indicating good overall diagnostic performance (**Figure 6**). The clinical usefulness was further shown by Fagan’s nomogram. A prior test probability of 20% indicated that a positive ^68^Ga-Pentixafor PET/CT result increased the post-test likelihood to 50%, whereas a negative result decreased the likelihood to 7% (**Figure 7**).

## Discussion

In this meta-analysis, 12 studies met the eligibility criteria, and 11 studies were included in the primary pooled analysis after exclusion of Hu et al. in 2023 (17) as an outlier. The pooled results showed that ^68^Ga-Pentixafor PET/CT had good AVS-referenced diagnostic performance for identifying unilateral PA. Most included studies showed a moderate to low risk of bias, and publication bias was unlikely according to Deeks’ funnel plot analysis.

The pooled sensitivity was 0.75 (95% CI: 0.67–0.82), indicating that the imaging modality correctly identified 75% of patients with unilateral PA. This moderately-high sensitivity is clinically relevant since many patients that would be considered for a potentially curative adrenalectomy would be identified correctly and would not be overlooked by the imaging procedure.

The pooled specificity was found to be 0.81 (95% CI: 0.74–0.87), indicating a strong capability to accurately identify patients who do not have unilateral disease. In practical terms, this means that ^68^Ga-Pentixafor PET/CT may reduce the chance of mistakenly identifying patients with bilateral disease as candidates for surgery, thereby reducing the risk of unnecessary adrenalectomy in patients who are unlikely to benefit from surgery. Additionally, the PLR was calculated at 4.05 (95% CI: 2.90–5.65), implying that patients with unilateral disease were approximately four times more likely to receive a positive result on ^68^Ga-Pentixafor PET/CT compared to those who do not have unilateral disease. A PLR > 4 is generally considered to provide moderate to high diagnostic value, increasing post-test probability to a clinically actionable level when pre-test probability is intermediate (34). The NLR was 0.31 (95% CI: 0.23–0.41), which implies that a negative test result reduced the likelihood of unilateral PA to approximately one-third of the pre-test probability (34).

From a clinical perspective, a low NLR suggests that ^68^Ga-Pentixafor PET/CT may help reduce the probability of unilateral PA. This capability may help clinicians avoid unnecessary surgical interventions in patients with negative imaging findings. The DOR was found to be 12.59 (95% CI: 7.21–22.01), which combined both sensitivity and specificity into a single measure. A DOR >10 is generally considered indicative of good test performance. The relatively high DOR observed in this study suggests that ^68^Ga-Pentixafor PET/CT consistently distinguished between patients with and without unilateral PA across various clinical settings and populations. Additionally, the AUC was 0.86. An AUC between 0.80 and 0.90 indicates good discriminatory ability, suggesting that ^68^Ga-Pentixafor PET/CT performs well across different diagnostic thresholds and pre-test probabilities. This is particularly relevant in real-world clinical environments, where pre-test probabilities can vary based on patient selection and the experience of institutions with adrenal imaging or AVS.

These findings suggest that ^68^Ga-Pentixafor PET/CT may help refine preoperative decision-making by reducing inappropriate surgical referral in selected patients, although prospective outcome-validated studies remain necessary (8). Nonetheless, additional prospective trials are needed to compare its diagnostic performance with AVS-based lateralization in different clinical settings and in different patient populations. Studies should evaluate concordance rates with internationally accepted diagnostic criteria and define situations in which PET/CT may provide additional clinical value.

Notably, the diagnostic performance of ^68^Ga-Pentixafor PET/CT in challenging clinical scenarios, such as for patients with borderline lateralization indices on AVS or those presenting with a non-functioning contralateral adrenal nodule on imaging, where both biochemical and imaging findings are inconclusive, warrants further evaluation. To address these knowledge gaps, carefully designed prospective head-to-head comparisons are required to further define the role of ^68^Ga-Pentixafor PET/CT in routine clinical practice (35). In addition, the combination of ^68^Ga-Pentixafor PET/CT with other advanced functional imaging techniques, such as PET/MRI hybrid modalities or radiomics-based pattern analysis, may substantially increase its sensitivity and anatomical resolution, especially for small or atypically localized aldosterone-producing lesions (36). In addition, prospective studies are also required to clarify the relationship between presurgical ^68^Ga-Pentixafor uptake and postsurgical clinical and biochemical cure rates and prognostication. Finally, to further unravel the molecular mechanisms underlying CXCR4 overexpression in aldosterone-producing tumors, translational studies investigating the pathophysiological basis of variability in radiotracer uptake may further elucidate the underlying tumor biology and open new perspectives in targeted therapy. Together, these research streams may in turn support the role of ^68^Ga-Pentixafor PET/CT as a diagnostic tool and potentially as a tool for future targeted management of primary aldosteronism.

### Limitations

This study has several limitations. First, AVS was used as the reference standard, but AVS does not represent a perfect clinical or pathological truth standard, and not all patients in the included studies had surgical, pathological, or postoperative outcome confirmation. Thus, the reported diagnostic performance mainly reflects agreement with AVS-based lateralization. Second, the number of included studies was small, and most studies came from limited geographic regions, which may limit the generalizability of the findings. Third, the criteria for positive ^68^Ga-Pentixafor PET/CT varied among studies, and a unified SUVmax cut-off could not be determined from the available data.

## Conclusions

This meta-analysis shows that ^68^Ga-Pentixafor PET/CT has promising AVS-referenced diagnostic performance for identifying unilateral PA, especially when AVS is inconclusive or unavailable. Its specificity facilitates the identification of unilateral disease and has the potential to inform surgical decision-making. Accurate localization may minimize the risk of unnecessary adrenalectomy in patients with bilateral or unclear disease and contribute to better informed treatment planning. Further prospective studies with surgical, pathological, biochemical, and clinical outcome validation are needed before ^68^Ga-Pentixafor PET/CT can be considered a replacement for AVS (37, 38).

## Data Availability

The original contributions presented in the study are included in the article/supplementary material. Further inquiries can be directed to the corresponding author/s.
